# Efficacy and safety comparison of esketamine-propofol with nalbuphine-propofol for upper gastrointestinal endoscopy in children: a multi-center randomized controlled trial

**DOI:** 10.3389/fped.2023.1126522

**Published:** 2023-06-27

**Authors:** Xiaosu Zheng, Jinjin Huang, Sisi Wei, Yingying Tao, Yang Shen, Yanting Wang, Pan He, Mazhong Zhang, Ying Sun

**Affiliations:** ^1^ Department of Anesthesiology, Shanghai Children’s Medical Centre, School of Medicine, Shanghai Jiao Tong University, Shanghai, China; ^2^ Department of Anesthesiology, The Children’s Hospital, Zhejiang University School of Medicine, National Clinical Research Center for Child Health, Hangzhou, China; ^3^ Department of Anesthesiology, Shanghai Children's Hospital, School of Medicine, Shanghai Jiao Tong University, Shanghai, China

**Keywords:** esketamine, nalbuphine, painless upper gastrointestinal endoscopy, children, propofol

## Abstract

**Background and Aims:**

Anesthetics such as propofol, esketamine and nalbuphine are used during the upper gastrointestinal endoscopy to achieve and maintain the desired sedation level. The aim of the study was to evaluate the effectiveness and safety of propofol-nalbuphine and propofol-esketamine in children.

**Methods:**

A multi-centered study was performed at three tertiary class-A hospitals. Children between 3 and 12 years old undergoing diagnostic painless upper gastrointestinal endoscopy were included and randomly divided into esketamine or nalbuphine group to estimate the primary outcome of successful endoscope insertion. The patients were given esketamine 0.5 mg/kg and propofol 2 mg/kg intravenously in esketamine group, with nalbuphine 0.2 mg/kg and propofol 2 mg/kg in the nalbuphine group. The primary outcome was success rate for the first attempt of endoscope insertion in each group. Secondary outcomes included the safety of both anesthesia regimens and gastroenterologist's satisfaction. We used the Face, Leg, Activity, Cry and Consolability (FLACC) scale to evaluate the level of pain before and during the procedure and the Pediatric Anesthesia Emergence Delirium (PAED) scale to assess the level of agitation and delirium after awakening from anesthesia.

**Results:**

Among 246 patients, 200 were randomly included in the final intention-to-treat analysis, with 100 patients in each group. The success rate for the first attempt of endoscope insertion in the esketamine group was higher than the nalbuphine group (97% vs. 66%; *P *< 0.01). The heart rate and mean arterial pressure after intraoperative administration in the esketamine group were higher than those in the nalbuphine group, while the delirium incidence during awakening was higher in esketamine group (all *P *< 0.05).

**Conclusion:**

The success rate for the first attempt of endoscope insertion of children undergoing upper gastrointestinal endoscopy in the esketamine group was higher than the nalbuphine group, propofol-related hemodynamic changes were reduced accordingly, while the incidence of esketamine-related adverse effects could be high.

**Clinical Trial Registration:**

Chinese Clinical Trial Registry: ChiCTR2000040500.

## Introduction

Endoscopy is the gold standard for diagnosis and treatment of most gastrointestinal diseases, and the last several years have witnessed an upsurge of gastrointestinal endoscopy under deep sedation/anesthesia in varies countries and regions to mitigate pain and increase comfort of patients (1–3). The sedation services are peculiarly crucial for children, for they always show the poor ability in tolerance and cooperation (4).

However, children's features of increased oxygen consumption, high basal metabolic rate, vulnerable airway and immature thermoregulation lead to increased risk of hypoxemia during sedation. In addition, anesthetic medication used and the left lateral decubitus position during the endoscopy procedure also increase the risk of hypoxemia (5, 6). Besides, deep sedation/anesthesia is associated with the increased risk of other adverse events, such as hypotension, hypertension and arrhythmia. The management of those adverse events is modifiable risk factors controlling, and for anesthetists the mainstay management is anesthetics medication or dosage adjustment.

Sedatives and analgesics play important roles in the alleviation of procedure-related discomfort and pain during outpatient surgery. Propofol is the most commonly used intravenous anesthetic, with the feature of short-acting, short half-life, lack of analgesia and minimized residual effects (7). Propofol's anesthetic effects is exerted as an allosteric potentiator and agonist of the gamma-aminobutyric acid type A (GABAA) receptor, by potentiating the central inhibitory gamma-aminobutyric acid (GABA) neurotransmitter (8). There are some adverse drug reactions (ADRs) of propofol should be considered, such as pain on induction, hemodynamic instability as well as cardiovascular and respiratory depression in a dose-dependent way (9). Combining anesthetics could reduce both medication dosages and dose-related adverse events. Propofol's pharmacodynamic (PD) interactions with opioids tend to be highly synergistic, while the incidence of respiratory depression increases when used together (10).

Nalbuphine is an opioid receptor agonist-antagonist on *κ*-receptor in the cerebral cortex and on *μ*-receptors in the medulla, with the analgesic potency being equivalent to morphine and being especially suitable for visceral pain (11, 12). The antagonistic effect on the µ-receptor makes nalbuphine show the “ceiling effect” on analgesic effect, for which nalbuphine is used as the medium potency opioid medication. Nalbuphine also represents ceiling effect on respiratory depression at about 0.2–0.4 mg/kg, thus makes it an ideal option for outpatient surgery in children (13, 14). Compared with other opioids, nalbuphine showed a lower incidence of respiratory depression (15).

Esketamine could be another potential alternative used in pediatric anesthesia. Esketamine was an N-methyl-D-aspartate receptor (NMDAR) antagonist, known for its dissociative anesthetic properties and strong analgesic effects. Esketamine has been widely used in pediatric anesthesia for the maintenance of children's spontaneous breathing, and its sympathomimetic properties also counteract the ADRs in respiratory and circulation systems caused by propofol (10, 16). It has been reported that esketamine was about two times more potent in anesthetic and analgesic effects compared with racemic ketamine, which may be partly due to its higher affinity with NMDAR, accordingly, esketamine has replaced ketamine in China for its smaller dosage and less dose-dependent side effects (17). Low-dose esketamine is now widely used to treat moderate to severe acute and chronic pain, while high-dose esketamine may lead to increased incidence of psychotomimetic and cognitive side effects, such as excitation and illusion (18).

Some studies have explained the advantages of combining esketamine with propofol and demonstrated the benefits when used together rather than respectively because of the smaller dosages of both anesthetics and the counteraction of the side effects with each other (16).

Therefore, the purpose of this multi-center, double-blinded randomized controlled study was to compare the efficacy of esketamine-propofol and nalbuphine-propofol in diagnostic upper gastrointestinal endoscopy in children. The second outcomes were the safety of both anesthesia regimens, including changes in hemodynamic parameters and the incidence of respiratory depression. Perioperative pain scores and gastroenterologist's satisfaction during the procedure were also collected.

## Methods

The multicenter, prospective, double-blind, randomized, two-arm trial was carried out during the period from 14 January 2021 to 10 June 2022 in 3 China hospitals, with the approval of the Institutional Review Board of Shanghai Children's Medical Centre, Shanghai (SCMCIRB-K2020098-3), Children's Hospital, Hangzhou, as well as Shanghai Children's Hospital, Shanghai, and after the registration in the Chinese Clinical Trial Registry (ChiCTR2000040500). The involved anesthesiologists all completed pre-training. The Helsinki Declaration was followed during the trial's administration.

### Study participants

Children between the ages of 3 and 12, ASA physical status (ASA-PS) II–III, and scheduled for a diagnostic gastrointestinal endoscopy under deep sedation/anesthesia without trachea intubation were enrolled. Exclusion criteria included patients who were obese (body mass index above 30 kg/m^2^), had a history of liver or kidney disease or dysfunction, required complex therapeutic procedures during the examination, had undertaken anesthesia within the previous seven days, and had an allergy to the medication being used. Legal guardians and children aged 8–12 were provided written informed consent during pre-anesthetic interviews. Information pertinent to this trial was thoroughly explained to children under the age of eight.

### Study procedure

Before the procedure, all patients were fasted for at least 6 h. About 5 min before anesthesia, children in the holding area were given 10 ml dyclonine 1% mucilage (manufactured by Yangtze River Pharmaceutical Group, China) orally for local anesthesia. The non-invasive blood pressure and pulse oximetry monitors were positioned when patients entered the operating room and lay on the operating table in the left lateral position, after which they were given i.v. esketamine (manufactured by Jiangsu Hengrui Pharmaceutical Co., Ltd., China) or nalbuphine (manufactured by Hubei Humanwell Pharmaceutical, China) in about 30 s, followed by propofol (manufactured by Fresenius Kabi Austria GmbH, Austria) in 30–60 s at a constant speed, according to protocol. A trained anesthesiologist prepared medication, then the anesthesia assistant re-examined the drug and doses. Esketamine and nalbuphine were both diluted into 10 ml syringes, thus the esketamine concentration was 5 mg/ml, and the nalbuphine concentration was 2 mg/ml, with each child receiving 0.1 ml/kg of medication.

After administration, the endoscopic examination was completed in one minute by a trained doctor. Patients were transported to the postanesthesia care unit (PACU) after the examination was finished, where the researcher documented data for at least 30 min until the patients' reawakening, with Modified Observer's Assessment of Alertness/Sedation Scale (MOSSA) score >4. A nasal cannula was used to deliver 2–3 L/min of supplementary oxygen during the procedure. Another anesthesiologist nearby was available for emergency assistance.

Children were released from PACU when their modified Aldrete score was greater than 9 and their vital signs were stable and normal. The patients’ follow-up was finished after 24 h, and the complications were documented.

### Observation indices

The demographic data and the procedure-related information, such as insertion success or failure, examination time (from endoscope insertion to the end of the examination), awakening time (from administration to the patients’ awakening), gastroenterologist and patient's satisfaction were collected and recorded.

Throughout the procedure, the heart rate (HR) and pulse oxygen saturation (SpO_2_) were continuously monitored, and the mean blood pressure (MBP) was measured at 1-minute intervals. All vital signs were recorded at the following six time points: on the examining table ready for anesthesia induction (T0; baseline), on finishing anesthesia induction (T1), at the time of endoscope insertion (T2), at the time of endoscopy examination ended (T3), arrived at PACU (T4) and discharge from PACU (T5).

During the study period, the following cardiorespiratory complications and other perioperative adverse events were noted: respiratory depression (SpO_2_ 93% for more than 10 s), hypotension (20% decrease from baseline in mean blood pressure), bradycardia (20% decrease from baseline in heart rate), dizziness, laryngospasm, vomiting, nausea, pruritus, headache, regurgitation, diplopia, delirium, hallucinations. Additionally recorded were emergency scenarios requiring assistance with ventilation.

A score of 6 or above indicates significant pain on the Face, Leg, Activity, Cry, and Consolability (FLACC) scale, which was used to measure awakening pain. In order to assess emergence delirium, the Pediatric Anesthesia Emergence Delirium (PAED) scale was applied, with a score of 10 or higher indicating delirium and agitation during the awakening period.

### Statistical analysis

The primary aim of the study was to compare the success rate of endoscope insertion in two groups, with success defined as patients showing no reactions to the endoscope during the first attempt (such as severe cough or movement or other adverse events). The second outcomes were cardiorespiratory complications and perioperative adverse events.

Randomization was finished by computer-generated random numbers concealed in opaque sequentially numbered envelopes. The investigators, pediatric patients and their guardians, the endoscopists as well as anesthesiology were blinded to the group allocation.

Outcome data were analyzed in the intention to treat (ITT) population. The success rate of esketamine 0.5 mg/kg combined with propofol was 0.85 in the pilot study, while it was 0.65 in nalbuphine group. With a significance level of 0.05 (*α* = 0.05) and a power of 90% (*β* = 0.10), the sample size required for each group is at least 94, according to PASS software (power analysis and sample size software, vision 11.0.7). Given the possibility of post-recruitment attrition, the two groups will each have 100 children.

The Shapiro-Wilk test was used to assess normal distribution of data. Continuous variables were expressed as mean ± standard deviation, and continuous normally distributed data were analyzed using one-way ANOVA, with the Bonferroni method used for group comparison tests. For continuous variables, repeated measures of ANOVA design were used to test two-way interactions (group and time effects). The Dunnett T3 method was used to compare nonnormally distributed data. The chi-square test was used to analyze categorical variables, which expressed as percentages.

SPSS 23.0 for Windows (SPSS Inc., USA) was used for statistical analysis. Statistical significance was defined as a *P*-value of less than 0.05.

## Results

**Figure 1:**
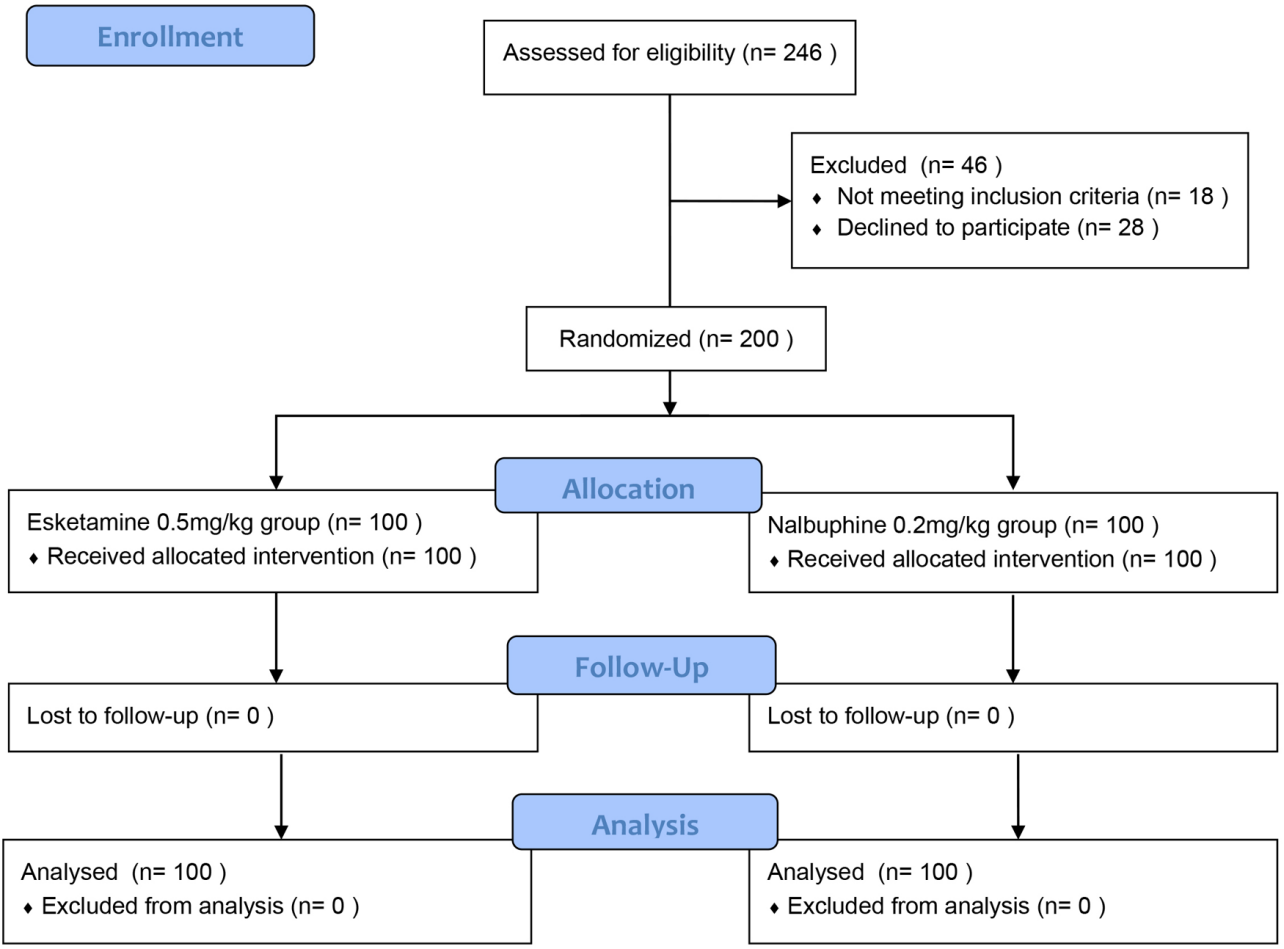
CONSORT diagram of participants randomised.

Totally 246 children were initially recruited and randomized, according to the ITT principle, of whom 200 patients were divided into two groups and analyzed in the final analysis, including 100 patients in the esketamine group and 100 children in the nalbuphine group, 28 patients or their guardians refused to sign informed consent, and 18 patients didn't meet the inclusion criteria (Figure 1).

**Figure 2:**
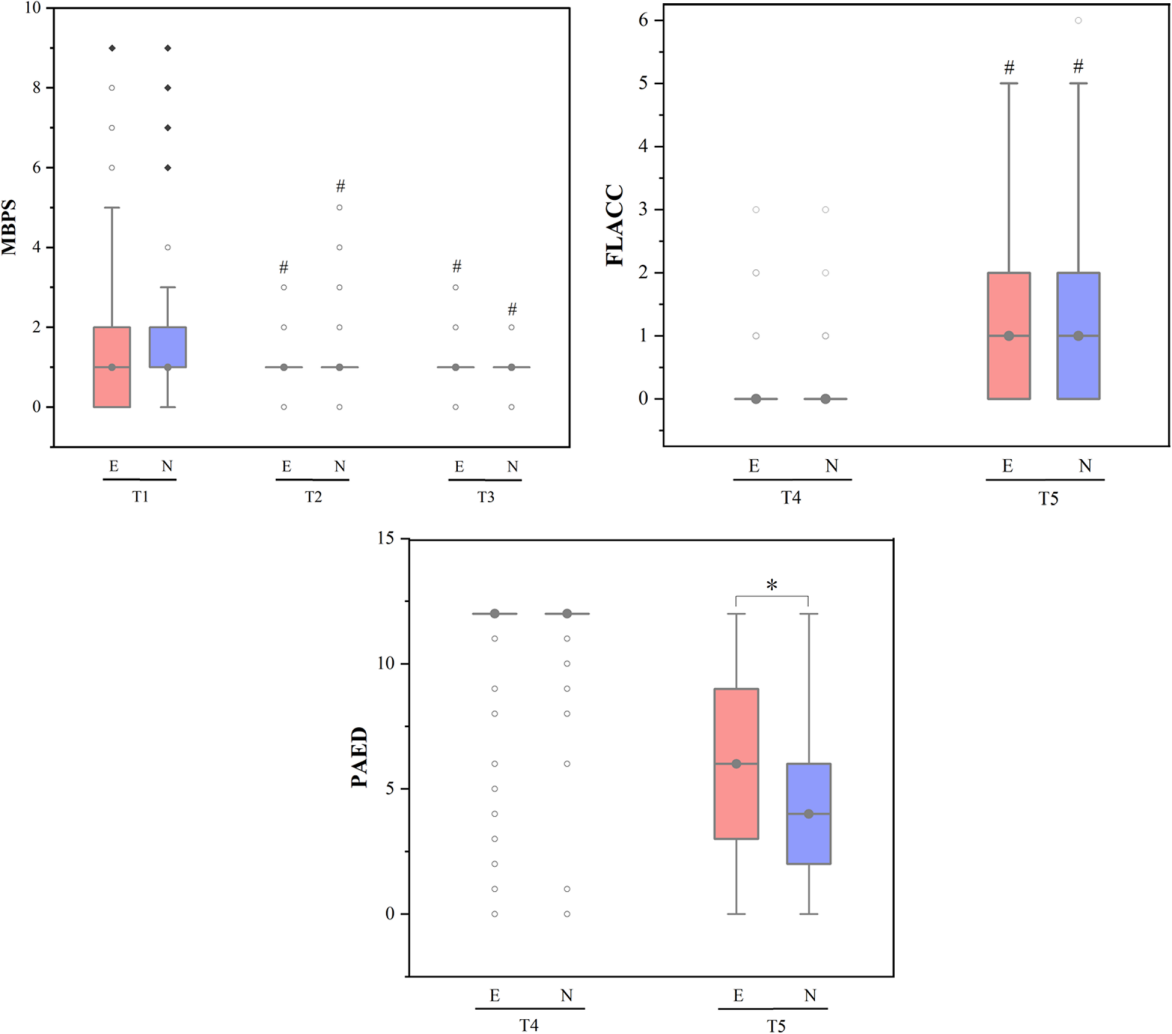
MBPS, FLACC and PAED scores across different study time points. Data was represented by the median (interquartile interval), the vertical line represented 1.5 times the interquartile spacing, ○ represented abnormal value and ◆ represented extreme value; MBPS, modified behavioral pain scale, FLACC, face, legs, activity, cry, consolability scale, PAED, pediatric anesthesia emergence delirium; E, esketamine group, N, nalbuphine group; Ready for anaesthesia induction (T0; baseline), on finishing anaesthesia induction (T1), endoscope insertion (T2), endoscopy examination ended (T3), arrived at postanaesthesia care unit (PACU) (T4) and on discharge from PACU (T5). ^#^*P*-value <0.05 compared with baseline (T0/T4) within group. *P-value <0.05 compared between groups.

### Primary outcome

There were no significant differences between two groups in patients’ demographics and procedure factors as listed in Table 1 (*P* > 0.05). Of the 100 children in the esketamine group, 97 were successful in the endoscope insertion, and of the 100 patients in the nalbuphine group, 66 were successful. The success rate of the endoscope insertion in esketamine group was significantly higher than nalbuphine group (97% vs. 66%, *P* < 0.05), and the endoscopist satisfaction was also better in esketamine group.

**Table 1:** Patients demographics.

	Esketamine 0.5 mg/kg Group (*n* = 100 group)	Nalbuphine 0.2 mg/kg Group (*n* = 100 group)	*P*-value
Age (years)	8.4 ± 2.1	8.2 ± 1.9	0.549
Body mass index (kg/m^2^)	16.3 ± 2.5	16.3 ± 2.9	0.855
Gender (male: female)	48:52	60:40	0.098
Examination time (min)	4.5 ± 2.0	4.4 ± 2.0	0.817
Awakening time (min)	15.7 ± 7.9	14.3 ± 6.9	0.200
Symptoms			
Abdominal pain	82 (82%)	78 (78%)	
Vomiting	11 (11%)	10 (10%)	
Gastritis	11 (11%)	17 (17%)	
Others	11 (11%)	14 (14%)	
Physician satisfaction (very/quite/generally/not)	81/15/4/0	57/19/15/9	<0.01
Patient satisfaction (very/quite/generally/not)	90/7/0/3	82/10/4/4	0.169

Data are presented as mean ± SD, except for gender, symptoms, physician and patient satisfaction as number. A value of *P *< 0.05 was considered significant.

### Secondary outcome

There were no significant differences in heart rate and mean arterial pressure between the two groups at baseline (both *P* > 0.05, Table 2). The heart rate of children at T1, T2, T3, T4 and T5 in the esketamine group was significantly higher than nalbuphine group (all *P* < 0.01). In esketamine group, compared with the baseline, HR of children increased significantly at T1, T3, T4 and T5 (both *P* < 0.05). In nalbuphine group, heart rate was significantly lower at T2, T3, T4 and T5 than baseline value (all *P* < 0.01). The mean arterial pressure of the two groups showed different trends over time after finishing administration, with a significant decrease at T1 time point in both groups compared with baseline (both *P* < 0.05) and significantly higher values at T1, T2 and T3 in the esketamine group compared with the nalbuphine group (all *P* < 0.01), and in nalbuphine group, the mean arterial pressure at T1, T2 and T3 was significantly lower than the baseline value (all *P* < 0.05).

**Table 2:** Changes in heart rate (HR) and mean blood pressure (MBP) across different study time points.

	Esketamine 0.5 mg/kg Group (*n* = 100 group)	Nalbuphine 0.2 mg/kg Group (*n* = 100 group)	*P*-value
Heart rate (beat/min)			
T0	93.9 ± 15.8	95.1 ± 16.1	0.591
T1	99.6 ± 13.2**	93.1 ± 14.1	<0.01*
T2	96.2 ± 13.3.0	88.8 ± 15.1**	<0.01*
T3	102.1 ± 16.5**	90.2 ± 14.8**	<0.01*
T4	98.5 ± 13.3**	87.4 ± 12.9**	<0.01*
T5	98.5 ± 15.5**	89.0 ± 12.9**	<0.01*
Mean arterial pressure (mmHg)			
T0	78.7 ± 12.0	79.3 ± 10.7	0.705
T1	75.4 ± 11.1**	66.5 ± 10.5**	<0.01*
T2	77.0 ± 14.4	65.4 ± 13.9**	<0.01*
T3	79.6 ± 12.8	64.5 ± 11.3**	<0.01*

Data are presented as mean ± SD. A value of *P *< 0.05 was considered significant. Ready for anaesthesia induction (T0; baseline), on finishing anaesthesia induction (T1), endoscope insertion (T2), endoscopy examination ended (T3), arrived at postanaesthesia care unit (PACU) (T4) and on discharge from PACU (T5).

* *P*-value <0.05 compared between groups.

** *P*-value <0.05 compared with baseline (T0) within group.

As shown in Figure 2, at T2 and T3, the MBPS (modified behavioral pain scale) scores of the two groups were significantly lower than the baseline value (all *P* < 0.05). At T5, the FLACC (Face, Legs, Activity, Cry, Consolability Scale) scores of the two groups were significantly higher than that at T4 (both *P* < 0.05). The PAED (Pediatric Anesthesia Emergence Delirium) scores of the two groups were higher at T5 than T4, (both *P* < 0.05), and at T5, the PAED score of children in esketamine group was significantly higher than that in nalbuphine group (*P* < 0.05).

During the examination, respiratory depression happened in 8 children in the esketamine group, and occurred in 6 children in the nalbuphine group (Table 3). During the recovery period, 8 children in the esketamine group reported diplopia, with no patients in the nalbuphine group showing the problem (*P* < 0.05). There was no significant difference in the incidence of adverse reactions such as dizziness, diplopia and vomiting between the two groups (all *P* > 0.05). All the adverse reactions were self-limiting.

**Table 3:** Adverse events.

	Esketamine 0.5 mg/kg Group (*n* = 100 group)	Nalbuphine 0.2 mg/kg Group (*n* = 100 group)	*P*-value
Intra-examination			
Respiratory depression	8 (8%)	6 (6%)	0.579
Awakening period			
Respiratory depression	2 (2%)	1 (1%)	1.000
Visual disturbance	8 (8%)	0 (0%)	0.012*
Dizziness	6 (6%)	1 (1%)	0.124
Itch	0 (0%)	8 (8%)	0.012*
Nausea	0 (0%)	3 (3%)	0.245
Headache	1 (1%)	2 (2%)	1.000
24 h after examination			
Dizziness	0 (13.04%)	1 (17.39%)	1.000
Nausea	1	3	0.614
Vomit	5 (4.35%)	10 (0.00%)	0.179

Data are presented as number. Hypotension was defined as more than 20% decrease in MBP when compared to baseline. Respiratory depression was defined as oxygen saturation less than 93% and lasting for at least 10 s. Visual disturbances included diplopia inability to see objects, etc. *A value of *P *< 0.05 was considered significant between groups.

## Discussion

The current study established that esketamine-propofol can be better than nalbuphine-propofol for the better deep sedation/anesthesia effect, superior hemodynamic profile and higher level of endoscopist satisfaction. However, the administration of esketamine increased the risk of medication related ADRs such as agitation on resuscitation.

In our study, the successful rate of the first insertion was 97/100 in the esketamine group and 66/100 in the nalbuphine group. The nalbuphine group had a lower success rate for endoscope insertion than the esketamine group did, but the clinical effect was nevertheless established.

There has been a lack of relevant dosage research of nalbuphine with propofol in children undergoing outpatient surgery. Borgeat et al. hypothesized that 0.1 mg/kg nalbuphine during anesthesia induction could lower the frequency of spontaneous movement generated by 3 mg/kg propofol (19). Chen et al. investigated that the ED95 of nalbuphine in painless induced abortion is 0.128 mg/kg (20). While in 2022, Tang et al. demonstrated that the nalbuphine dosage of 0.15 mg/kg was better than 0.1 mg/kg, as the nalbuphine dose increased from 0.1 mg/kg to 0.15 mg/kg, the ED95 of propofol in adults decreased significantly from 2.759 mg/kg to 2.243 mg/kg, with the lower incidence of hypotension and the shorter recovery time (21). Li et al. concluded that the ED95 of nalbuphine combined with propofol in adults was 0.162 mg/kg (11). However, Deng et al. believed that there was no significant difference in analgesic effect between sufentanil 0.1 *μ*g/kg and nalbuphine 0.1 mg/kg, 0.15 mg/kg and 0.2 mg/kg combined with propofol respectively, thus they demonstrated that the optimal nalbuphine dosage range was 0.1–0.2 mg/kg (22). We ultimately chose a nalbuphine dosage of 0.2 mg/kg considering about the analgesic requirement and based on the clinical experience.

Ketamine is a phencyclidine derivative consisting of two optical isomers named S- and R-ketamine, it was first synthesized in 1962, then conducted on volunteers in 1964, and was approved to use clinically in1970 (23, 24). Esketamine has played a versatile role in pediatric anesthesia because of the various routes of administration as well as for the unique feature of “dissociative anesthesia”, strong analgesia, minimized inherent respiratory depression, hemodynamic stability and intrinsic sympathomimetic activity (17, 25). Besides, it also exerts diversity effects of anti-inflammatory, antihyperalgesia, neuroprotective and antidepressant (26). In 2019, Wang et al. compared 0.5 mg/kg esketamine and 1 mg/kg racemic ketamine in painless upper gastrointestinal endoscopy, and evaluated that esketamine, instead of larger dose of ketamine, could be used in regular sedation or anesthesia (16). According to our previous study, the ED50 of propofol combined with 0.5 mg/kg esketamine was 1.8 [95%CI, 1.1–2.4] mg/kg in children (27). Since there is a lack of dosage research on esketamine, we combined 0.5 mg/kg esketamine with propofol, and the results showed that more than 90% of the endoscope insertion was successful.

Unlike adults, hypoxemia, usually happened within 5 min after endoscope insertion, remained to be a major complication of procedural sedation or anesthesia in children, even up to 70%–80% according to some studies (28, 29). Propofol is one of the most common intravenous anesthetics clinically, while its lack of analgesic effect makes more propofol is needed for expected anesthetic depth during the outpatient surgery or some short surgery. Furthermore, the combining medication induces dosage-related ADRs of both drugs, for youngsters with frail circulatory and respiratory systems it could be important (30–32). Besides, children's poor oxygen reserves and high oxygen consumption also increase the risk of hypoxemia (5, 33). Given that the typical opioids such as fentanyl, remifentanil and sufentanil are also associated with respiratory depression, esketamine and nalbuphine were chosen in the study (10, 34).

In our research, the incidence of intraoperative respiratory depression was 9% in the esketamine group and 10% in the nalbuphine group, with no statistically significant differences between groups, which may be due to the characteristics of the two medications. Traditional opioids' respiratory depression is mostly mediated by µ receptor activation, whereas nalbuphine's distinctive partial µ receptor antagonism causes its respiratory inhibition to exhibit the “ceiling effect”, which causes nalbuphine's limited effect on respiratory system (22, 35, 36). Esketamine produces bronchodilation status and maintains the hypercapnic reflex, which results in relatively more conserved airway reflexes and less respiratory depression (10). Abusing 10 times the recommended dosage of nalbuphine in neonates resulted in prolonged sedation duration but no significant respiratory depression or failure, according to Schultz machata et al. (35).

The lower incidence of hypoxemia might be also associated to the preventive oxygen inhalation via nasal catheter given to children prior to induction, which can reduce the incidence of hypoxia to some extent. In our research, children having upper gastrointestinal endoscopy under anesthesia had a considerably decreased risk of hypoxia than that of Hayes et al., which was likely benefit from the preventive oxygen administered through nasal cannula (37). The oxygen inhaled through the nasal catheter employed in our study may have effects simillar to those of high flow nasal cannula (HFNC), a non-invasive respiratory support system that continuously delivers high flow gas for heating and humidifying. HFNC provides constant positive airway pressure and facilitates carbon dioxide release by reducing dead space, which has been shown to lower the incidence of hypoxia in researches for some adults. However, during upper gastrointestinal endoscopy with fentanyl and propofol, Klotz et al. found in 2020 that HFNC did not delay the onset of hypoxia or enhance respiratory system stability when compared to regular low-flow nasal oxygen (38).

The sympathetic nervous system properties of esketamine can counteract hypotension caused by propofol. According to our findings, children in the esketamine group had more stable hemodynamics, and aside from a brief drop at T1, the mean arterial pressure had no significant changes compared to the baseline value at any other time points; the mean arterial pressure in the nalbuphine group remained lower than the baseline value after administration. Children's heart rates varied between two groups. The heart rates in esketamine group exhibited an overall high trend from the end of administration to the recovery period, but in nalbuphine group, heart rates were significantly lower than the baseline value.

The findings of this study indicated that PAED scores at the time of awakening (T5) in the esketamine group was considerably greater than that in the nalbuphine group. It was congruent with the findings of Dalens et al., who reported that intravenous administration of small doses of ketamine or nalbuphine at the end of an MRI examination can reduce emergence agitation during the awakening period in children under sevoflurane, and nalbuphine had a better effect than ketamine (39). In addition to agitation during the awakening period, nystagmus was more common in children in the esketamine group than in the nalbuphine group. Likewise, there was no statistically significant difference in awakening time or the incidence of nausea and vomiting between the two groups.

As to inducing anesthesia, children in our study received local lidocaine spray in the throat, but some institutions utilized intravenous lidocaine. A meta-analysis of 1,707 clinical studies involving propofol sedation or anesthesia with intravenous or local lidocaine for gastrointestinal endoscopy was published in the British Journal of Anesthesiology in 2021, and they found that when intravenous injection or local lidocaine was employed as an auxiliary measure for propofol sedation, the discomfort following operation, the risk of vomiting events and unconscious movement were all decreased, and there was no influence on circulation or respiration (40). The researchers conducted a subgroup analysis of the intravenous and local anesthetic groups as well. The findings showed that intravenous lidocaine has advantages over local lidocaine, including the ability to reduce propofol dosage, boost endoscopist satisfaction, and speed up recovery in these patients.

There are still some limitations to our research. First and foremost, our study recruited children aged 3–12. The patients in the study were not divided by age. According to certain researches, age was an independent factor in the occurrence of adverse events after deep sedation or anesthesia during upper gastrointestinal endoscopy in children. As a result, additional age stratification study will be conducted in the future. Second, the most recent review and analysis results demonstrated that intravenous lidocaine can minimize propofol dosage, enhance endoscopists satisfaction and shorten recovery time, which were benefits that local lidocaine did not have. In this investigation, lidocaine was just administered as a local anesthetic. Third, there is currently a dearth of relevant data in children about the ceiling effect of nalbuphine in analgesia and respiratory depression, therefore more pharmacokinetic and pharmacodynamic researches should be finished to determine the more precise dose. Finally, our study only included children with ASA scores of I–III, the findings may not be applicable for other patients.

## Conclusion

Both esketamine 0.5 mg/kg or nalbuphine 0.2 mg/kg in combination with propofol can be safely and effectively used for pediatric upper gastrointestinal endoscopy. For the children in the group of esketamine 0.5 mg/kg combined with propofol 2 mg/kg, the success rate for endoscope insertion was higher, the total amount of propofol required was less, and the hemodynamics were more stable than in the group of nalbuphine 0.2 mg/kg combined with propofol 2 mg/kg, while the incidence of adverse effects such as agitation and diplopia in the esketamine group during awakening period was higher.

## Data Availability

The raw data supporting the conclusions of this article will be made available by the authors, without undue reservation.
